# Stress fracture of the pelvis and lower limbs including atypical femoral fractures—a review

**DOI:** 10.1007/s13244-014-0371-z

**Published:** 2014-12-02

**Authors:** Bernhard J. Tins, Mark Garton, Victor N. Cassar-Pullicino, Prudencia N. M. Tyrrell, Radhesh Lalam, Jaspreet Singh

**Affiliations:** 1 Department of Metabolic Medicine, Robert Jones and Agnes Hunt Orthopaedic and District Hospital, NHS Trust, Twmpath Lane, Oswestry, SY10 7AG UK; 2 Department of Radiology, Robert Jones and Agnes Hunt Orthopaedic and District Hospital, NHS Trust, Twmpath Lane, Oswestry, SY10 7AG UK

**Keywords:** Stress fracture, Insufficiency fracture, Atypical femoral fracture, Metabolic bone disease, Imaging

## Abstract

Stress fractures, that is fatigue and insufficiency fractures, of the pelvis and lower limb come in many guises. Most doctors are familiar with typical sacral, tibial or metatarsal stress fractures. However, even common and typical presentations can pose diagnostic difficulties especially early after the onset of clinical symptoms. This article reviews the aetiology and pathophysiology of stress fractures and their reflection in the imaging appearances. The role of varying imaging modalities is laid out and typical findings are demonstrated. Emphasis is given to sometimes less well-appreciated fractures, which might be missed and can have devastating consequences for longer term patient outcomes. In particular, atypical femoral shaft fractures and their relationship to bisphosphonates are discussed. Migrating bone marrow oedema syndrome, transient osteoporosis and spontaneous osteonecrosis are reviewed as manifestations of stress fractures. Radiotherapy-related stress fractures are examined in more detail. An overview of typical sites of stress fractures in the pelvis and lower limbs and their particular clinical relevance concludes this review.

*Teaching Points*

• *Stress fractures indicate bone fatigue or insufficiency or a combination of these.*

• *Radiographic visibility of stress fractures is delayed by 2 to 3 weeks.*

• *MRI is the most sensitive and specific modality for stress fractures.*

• *Stress fractures are often multiple; the underlying cause should be evaluated.*

• *Infratrochanteric lateral femoral fractures suggest an atypical femoral fracture (AFF); endocrinologist referral is advisable.*

## Introduction

Fatigue and insufficiency fractures of the pelvis and lower limb come in many guises. The common presentations are well known to radiologists yet can still pose diagnostic difficulties especially early after the onset of clinical symptoms.

This article concentrates on the pelvis and lower limbs as the most common sites for stress fractures.

Stress fractures in the immature skeleton are not included as this is beyond the scope of this article. Aetiology, pathophysiology and biomechanics in children differ significantly from those in adults.

This article reviews the mechanisms leading to stress fractures of bone in adults and how this is reflected in the imaging appearances.

Emphasis is put on some of the more difficult presentations of stress fractures; in particular atypical femoral shaft fractures, bone marrow oedema syndrome and radiotherapy-related stress fractures are discussed.

A brief overview of typical sites of stress fractures in the pelvis and lower limbs and their particular clinical relevance concludes this review.

## Aetiology

**Fig. 1:**
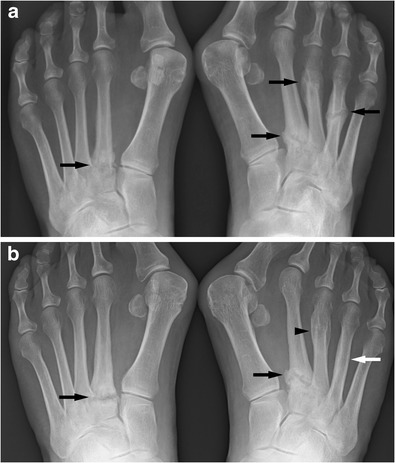
PA radiographs of the feet of a middle-aged female patient. The first radiograph (**a**) shows stress fractures in both feet in various stages of healing (black arrows). Symmetrical fractures at the base of the second metatarsals. Two years later (**b**) the fractures at the base to the second metatarsals have still not healed (black arrows); the fracture of the right fourth metatarsal has remodeled well and is now hard to appreciate (white arrow); the fracture of the right third metatarsal has healed (black arrowhead). Stress fractures are not rarely bilateral and symmetrical. Fractures at the base of a metatarsal heal less well than midshaft fractures

The first descriptions of stress fractures go back hundreds of years. Rib fractures due to chronic cough were described by Gooch as far back as 1733. Stress fractures in the foot were described by Breithaupt, a military physician, in 1855 (Fig. 1). He described painful focal forefoot swelling in military recruits after strenuous marches, the origin of the term march fracture. Very soon after the discovery of Röntgen-/X-rays Stechow described the appearance of march fractures on radiographs [1, 2].

Generally, stress fractures can be due to abnormal stress on normal bone or fatigue fractures, while insufficiency fractures occur because of normal stress on abnormal bone. The differentiation between a fatigue and an insufficiency fracture is not always easy, as both can occur at the same time in the same patient and fatigue as well as insufficiency can contribute to any fracture. The use of the term “stress fracture” relieves the radiologist from committing himself to a more specific diagnosis of a fatigue or an insufficiency fracture. It is often not possible to differentiate between the two without further information.

Colloquially the term stress fracture is also used instead of fatigue fractures. In this article stress fracture will be used in its original meaning including fatigue and insufficiency fractures.

The incidence of stress fractures in the general population is about 1 % while in runners it is up to 20 %. In patients with rheumatoid arthritis a prospective study found an incidence of 11.5 fractures per 100 patient years. More than 90 % of stress fractures affect the lower extremities, and this article will concentrate on the pelvis and lower limbs [3–5].

Once a patient has developed a stress fracture there is increased risk of further stress fractures in the same limb and the contralateral side. This can occur at the time of presentation or later on and is due to the fact that typically the stresses are similar in both limbs (i.e. in runners) [3, 4, 6].

**Fig. 2:**
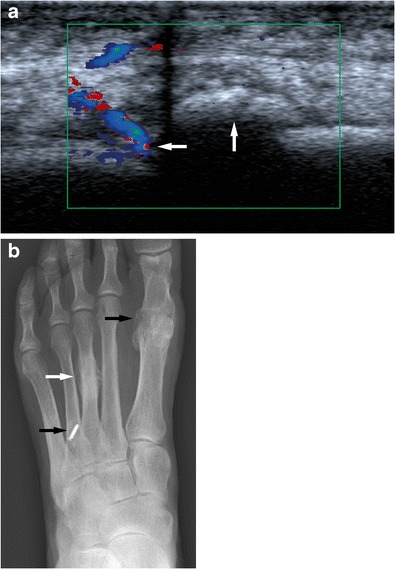
An elderly female attending for an ultrasound of the foot to assess forefoot pain. A longitudinal ultrasound image (left side is proximal) demonstrates partly ossified hypervascular callus (white arrows) of a metatarsal (**a**) suggestive of a healing metatarsal fracture. A PA radiograph of the left foot (**b**) shows evidence of previous surgery (white arrows) and a typical stress fracture of the left third metatarsal (white arrow). Previous surgery particularly of the feet is a risk factor for the development of stress fractures. Ultrasound is not the imaging modality of choice for the assessment of stress fractures but can incidentally show stress fractures

Fatigue fractures usually result from cyclic loading on bone that exceeds the bone’s natural repair capacity. Fatigue fractures most often occur in physically active individuals often after a sudden increase in activity. They mostly affect the lower extremities and they are disproportionately common in runners, dancers, military recruits and participants in any sport involving a significant amount of running and jumping. They also occur after orthopaedic surgery, especially lower limb and in particular foot surgery (Fig. 2). This is thought to be due to a combination of altered gait pattern and some bone loss due to a period of reduced activity after surgery, therefore involving an element of insufficiency type fracture. Limited physical fitness also plays a role [4, 6–8].

**Fig. 3:**
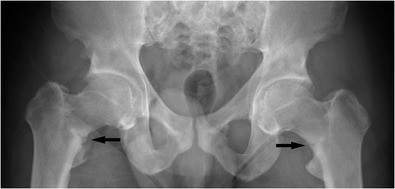
Adult patient with treated hypophosphataemic rickets. Abnormal bone texture in the pelvis and proximal femora with bilateral stress fractures of the femoral necks (black arrows). Abnormal bone texture predisposes to stress fractures

In insufficiency fractures the fractures occur in response to normal everyday activities, again typically activities with cyclical loading. The cause for insufficiency fractures is mechanically abnormally weak bone (due to low bone quality and mass) or impaired bone repair. Typically females with osteoporosis and older than 50 years of age are affected. Other underlying pathologies are metabolic problems such as glucocorticoid use or Cushing’s disease, vitamin D deficiency, rickets (Fig. 3), hyperparathyroidism, renal osteodystrophy and osteomalacia. Vitamin D deficiency may be due to many factors including inadequate exposure to sunlight, vitamin malabsorption (i.e. due to celiac disease, lactose intolerance, cystic fibrosis, small bowel resection, etc.), drugs inducing hepatic p450 enzymes (i.e., phenytoin, phenobarbital, rifampicin, etc.) and liver and renal disease. Some substances such as proton pump inhibitors can affect the absorption of vitamins and minerals and are increasingly suspected to cause or contribute to bone fractures [9, 10]. Patient behaviour-related causes are nicotine and alcohol abuse and anorexia. Low adult weight, white race, female gender and low bone density are further risk factors. Diseases associated with insufficiency fractures are rheumatoid arthritis, diabetes mellitus (abnormal bone quality and neuropathy), Paget’s disease, osteogenesis imperfecta, fibrous dysplasia, skeletal dysplasias and drugs such as sodium fluoride, methotrexate and etidronate and probably also bisphosphonates. An at least partly iatrogenic cause is radiotherapy. As in the fatigue fracture group a relatively sudden increase of activity levels is a risk factor for insufficiency fractures [3, 4, 6, 11]. If the bone density is known, the fracture risk can be determined using online tools [12].

## Pathophysiology and its relevance to imaging appearances

Any physical everyday activity leads to microfractures of bone, which are repaired by initial osteoclastic resorption followed by osteoblastic bone formation. In the context of normal activity and normal bone this is normal, physiological regeneration of bone. However the time lag between resorption and build-up is about 2 to 3 weeks. In cases of abnormally weak bone or an abnormally high microfracture rate or a delayed repair process, the microfractures can accumulate.

An additional repair process in cases of more extensive bone stress is a periosteal reaction where the periosteum begins to thicken and offers additional mechanical support. The development of a periosteal reaction has a similar lag time as the osteoblastic reaction.

**Fig. 4:**
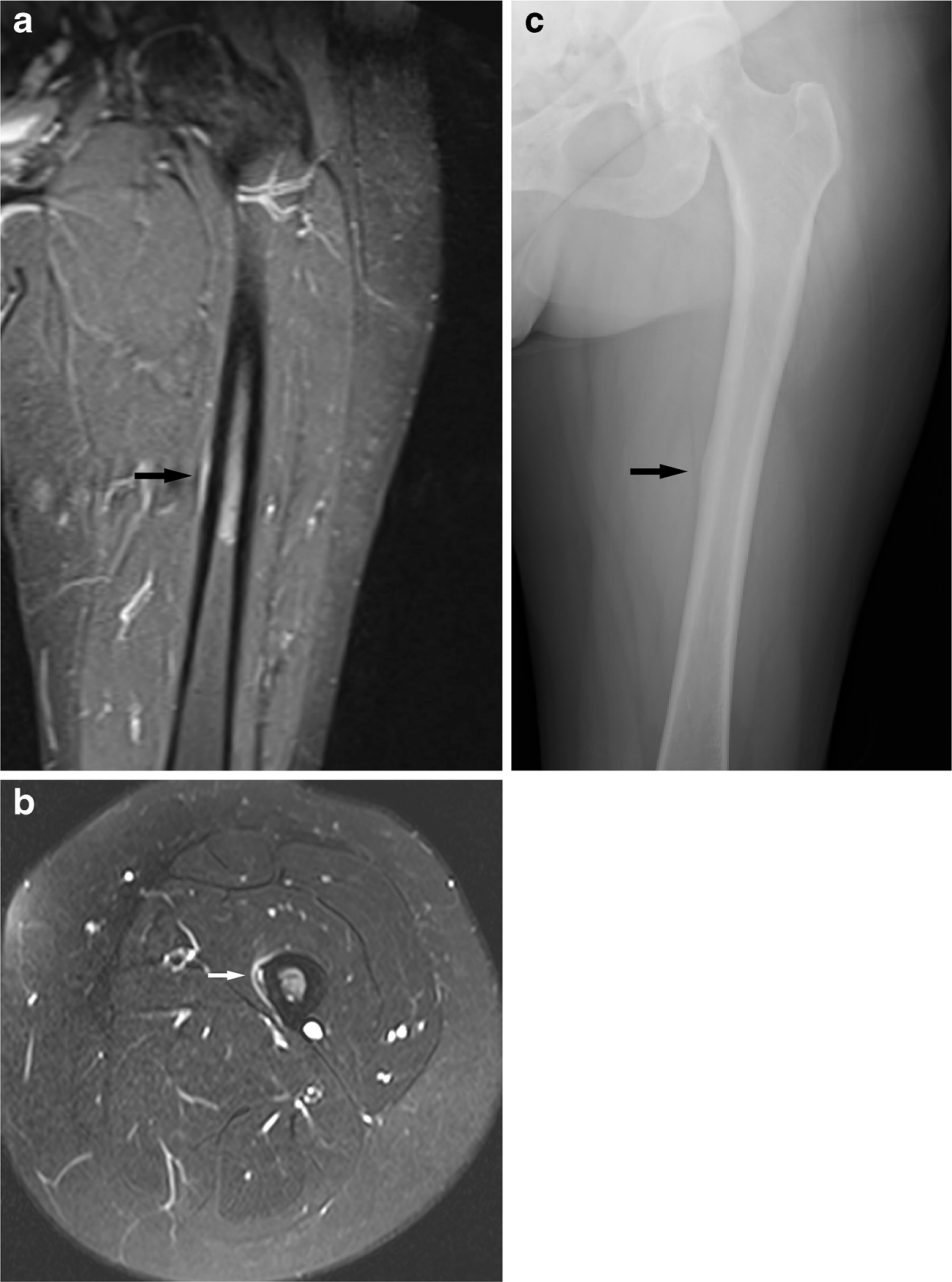
Typical stress lesion of the left midfemoral shaft in a middle-aged female. This is difficult to appreciate on the coronal images (**a**, short tau inversion recovery, STIR, image) and is just visible as subtle medial high fluid signal at the midshaft (black arrow). It is best seen on an axial proton density fat saturation (PD-FS) sequence (**b**) as a medial periosteal reaction and intracortical high fluid signal (white arrow). This was not visible on a radiograph taken at the same time but became radiographically visible 2 months later (**c**) as a subtle periosteal reaction medially (black arrow). Typical femoral stress fractures are located on the medial femoral shaft and have a broad-based bone reaction

Bone and periosteal reactions therefore do not occur until several weeks into the disease process. This is the reason for the delayed occurrence of radiographically visible changes in stress fractures. This also explains why in relatively acute stress fractures there is a cortical lucency without a periosteal reaction or callus formation. With the healing response a periosteal and often also endosteal bone reaction is seen. These changes are often very focal (Fig. 4) [4, 7, 13–16].

Most stress fractures occur in the pelvis and lower extremities simply by virtue of high mechanical loading of the pelvis and legs exacerbated by many sports. Low bone turnover particularly in the long bones of the leg (≤1 % of bone mass per year) favours the accumulation of microdamage eventually leading to overt bone failure / fracture [10].

Compressive stresses are generally better tolerated than tensile stresses; muscle strength and endurance (or rather the lack thereof) are also important contributors to stress lesions of bone. There is often an unhappy triad of a firstly new or different, secondly strenuous and thirdly repetitive activity [4, 10, 13].

The findings discussed above determine the imaging appearances. Acute stress fractures are often difficult to image radiographically unless cortical disruption with significant displacement has occurred. However a cortical radiolucency without a periosteal reaction or callus formation might be visible in the early phase of a stress fracture. On initial clinical presentation radiographs have a sensitivity of less than 50 % for stress fracture with some authors describing sensitivities as low as 10 % [4, 13, 17].

Similarly CT is more sensitive than radiography but not as sensitive as MRI or bone scintigraphy [16, 18].

The reactive changes in bone metabolism lead to the formation of granulation tissue, which can be visualised as increased fluid signal on MRI and hyperaemia in the early phases of the bone scintigraphy. Interestingly bone phase imaging does show increased uptake early on; the initial delayed osteoblastic reaction does not seem to impair the sensitivity of bone scintigraphy for the diagnosis of stress fractures. The main weakness of bone scintigraphy is the lack of specificity due to limited anatomical resolution and correlation and more importantly the inability to assess the significance of the findings. Foci of increased bone uptake in athletes have been found to be usually normal bone remodelling responses to training. Bone scintigraphy is not able to assess the amount of lysis and therefore fracture risk [17, 19, 20].

MRI is therefore the investigation of choice combining high sensitivity with high specificity. MRI shows increased fluid signal in medullary bone and, in cases of cortical and periosteal involvement, also in adjacent soft tissues. A sclerotic response may be visible as a low signal line in all sequences [4, 5, 13, 16, 21]. The use of intravenous (IV) contrast medium has not been found to be helpful in stress fractures and on the contrary might mask the fracture because of a signal increase of the otherwise low signal area in T1 weighting [22]. MRI is considered the gold standard for the imaging of stress fractures. The main drawbacks of MRI are availability and cost [4, 13, 23].

**Fig. 5:**
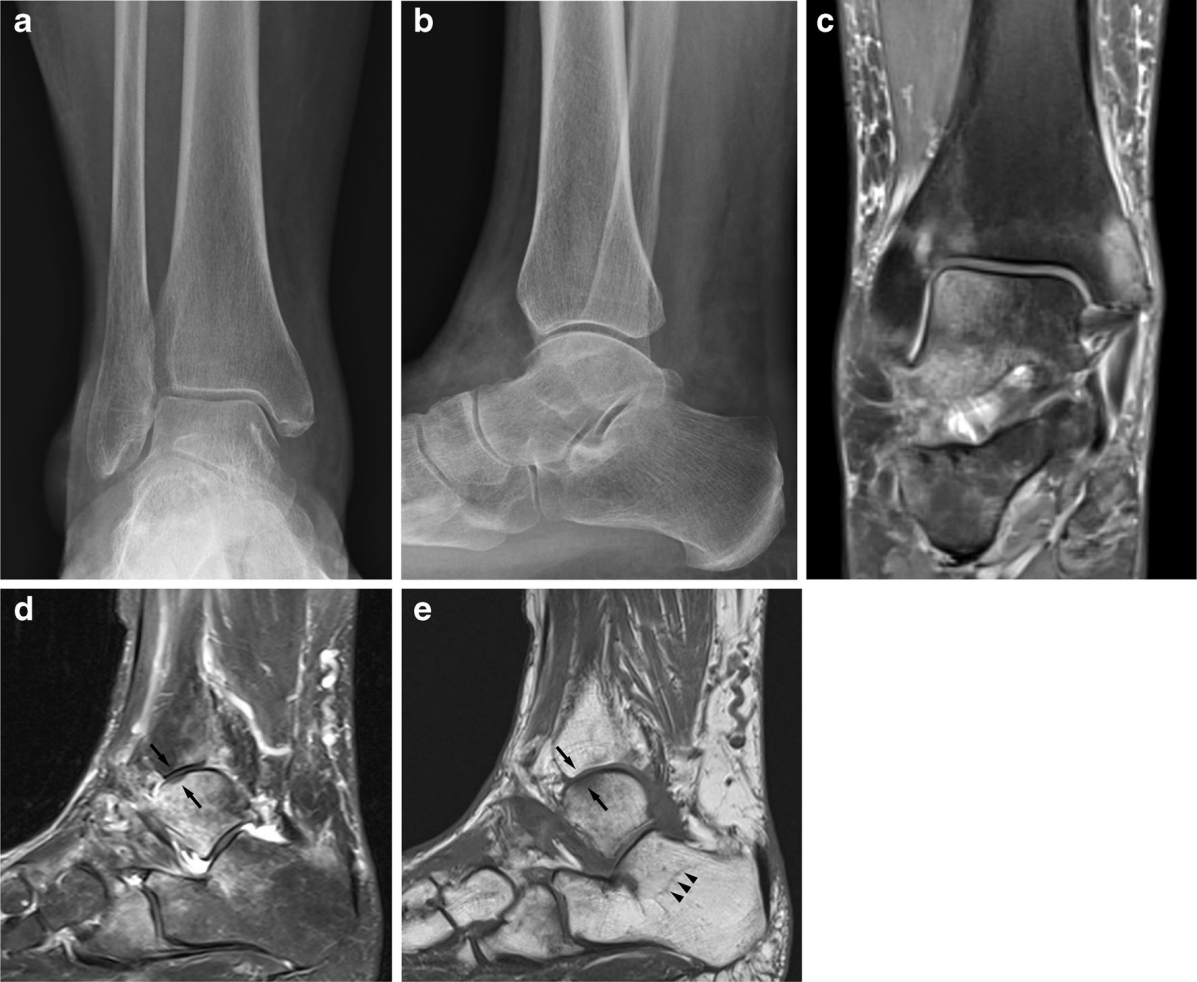
An elderly female patient with foot pain. AP (**a**) and lateral (**b**) radiographs of the ankle show osteopenia but do not show a reason for significant foot pain. Coronal (**c**) and sagittal (**d**) PD-FS sequences show significant bone marrow oedema-like changes in the medial malleolus, lateral ankle joint, calcaneus and navicular and cuboid. In the lateral talar trochlea there is focal low signal change in fluid sensitive (**d**) and T1w sequences (**e**) consistent with focal subchondral infraction (black arrows). The sagittal T1w sequences (**e**) demonstrate low signal lines in the cancellous bone indicating stress fracture lines in cancellous bone (arrowheads)

**Fig. 6:**
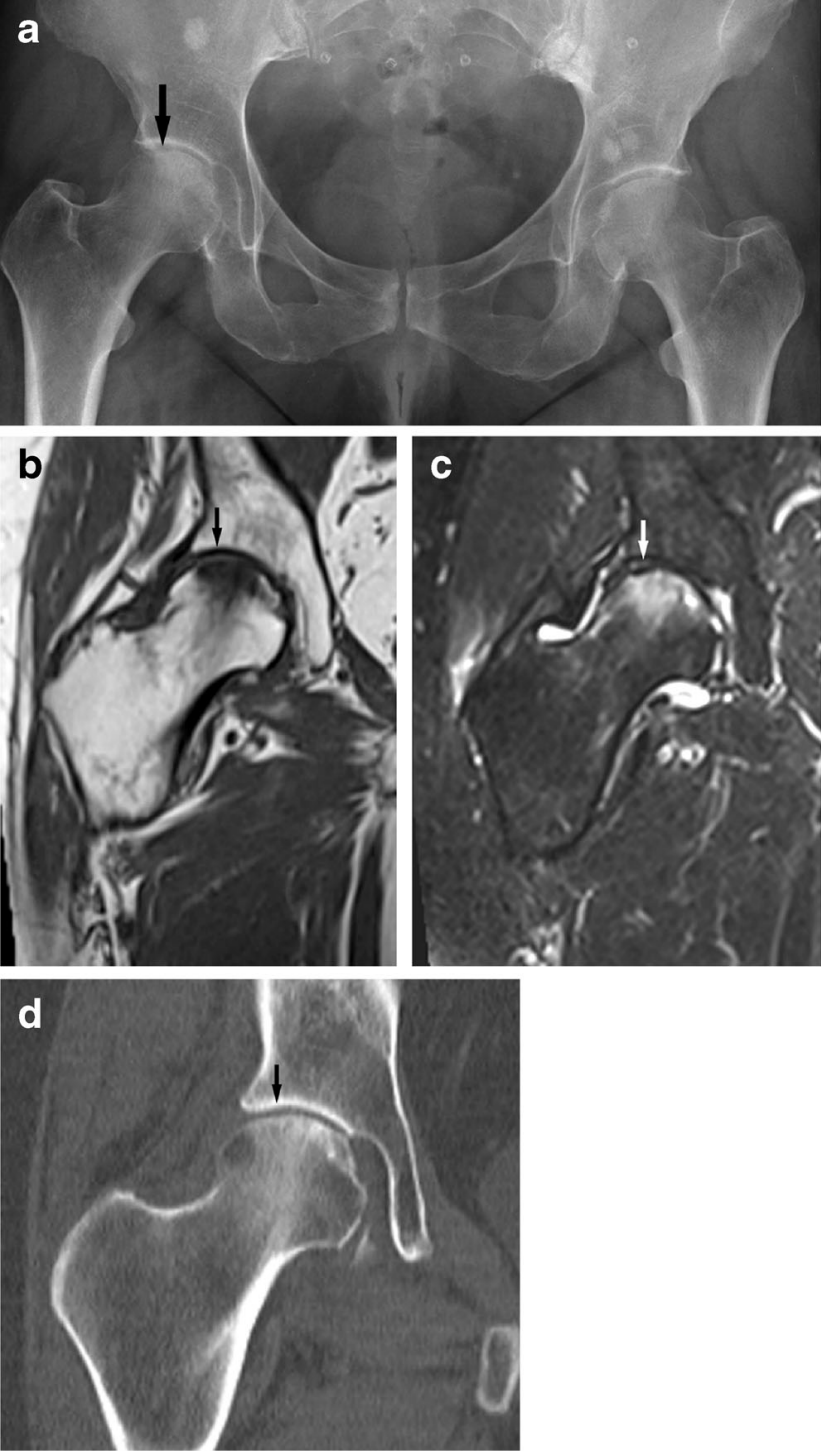
A 60-year-old female presenting with groin pain. An AP radiograph of the pelvis (**a**) shows subtle subchondral flattening of the femoral head suspicious for a stress (insufficiency) fracture (see arrow). Multiple bone islands are noted. Sclerosis and irregularity of the symphysis pubis again suggest bone stress. On radiography alone avascular necrosis also has to be considered as a cause of subchondral flattening; however the femoral head infraction is confirmed with MRI [coronal T1w (**b**) and T2-FS (**c**) images] (see arrows) and avascular necrosis (avn) is effectively excluded. This is confirmed with CT imaging (**d**). These lesions need specialist attention to prevent deterioration to rapidly destructive OA

If only cancellous bone is involved subtle blurring of trabecular margins and faint sclerosis may be visible radiographically. Stress fractures of cancellous bone are difficult to visualise on radiographs, and a sclerotic bone reaction is easier to appreciate on CT. Bone scintigraphy is a very sensitive but not very specific examination technique. MRI is best suited to demonstrate this pathology. MRI shows bone marrow oedema-like changes and sclerosis may also be visible (Figs. 5 and 6) [4, 7, 13–16].

The use of ultrasound in the detection of stress fractures has been described as far back as 1992 in a letter by Howard and colleagues [24]. Since then there have been further publications mainly describing incidental findings of stress fracture in the feet when performing ultrasound for the assessment of painful areas [25, 26]. The use of ultrasound in suspected metatarsal stress fractures has been assessed. Banal and colleagues found that in X-ray-negative and MRI-positive metatarsal stress fractures, ultrasound was positive in more than 80 % of cases, making it a low cost but limited alternative to MRI [23].

Sonographic findings suggestive of an early stress fracture are a hypoechoic, hypervascular rim around the cortex of a painful bone. This is due to a periosteal reaction. Soft tissue oedema may be visible. In later stages cortical irregularity or disruption and later still callus formation may become visible (Fig. 2) [25–27].

The high cost and limited availability of MRI has led to the development of guidelines to support clinical decision making. The US military has proposed to primarily use radiography, at the earliest 2 weeks after the onset of symptoms. If negative, the radiographs are repeated a further week later. If still negative an MRI is performed. The recommendation for US American family physicians also envisages the initial use of radiographs for suspected stress fractures. If negative, ideally MRI should be performed; if this is not feasible bone scintigraphy is advised [5].

The initial use of radiographs has the further advantage of being able to assess for other significant pathologies such as tumours, degenerative or inflammatory changes, malalignment or frank trauma, etc.

In the authors’ practice ultrasound is not routinely performed for the assessment of stress fractures although it is important to be aware of the imaging findings. The authors have encountered a number of patients referred for ultrasound examination with non-specific foot or ankle pain who were found to suffer from stress fractures.

## Clinical presentation

Fatigue fractures are typically associated with pain on exercise that abates with rest. As the severity of the stress injury to bone increases, the pain appears earlier in the exercise and takes longer to abate until even at rest some pain may persist. However stress-related bone changes shown on imaging examinations may not always be symptomatic and may disappear without ever having become symptomatic. In a study of distance runners 9/21 showed stress-related MRI anomalies of the tibiae [7, 13, 19–21].

In particular in the elderly or patients with other underlying pathologies such as rheumatoid arthritis, stress (here particularly insufficiency) fractures can cause significant morbidity, and early diagnosis and adequate treatment are very important to prevent permanent deterioration [3, 6].

Most stress fractures can be treated conservatively with rest or adjustment of exercise regimes, but in high-risk stress lesions or persistent non-healing ones surgical intervention may be appropriate.

## Special considerations

### Atypical femoral shaft fracture

More recently the phenomenon of the atypical femoral shaft fracture (or atypical femoral fracture, AFF) has become recognised. This describes the occurrence of femoral stress fractures affecting the femoral diaphysis (between the immediate subtrochanteric area to just proximal to the epicondyles) but with atypical features compared with a “typical” femoral shaft stress fracture.

The American Society for Bone and Mineral Research (ASBMR) has published the following criteria for the diagnosis of atypical femoral stress fracture [4, 28]:

- "To satisfy the case definition of AFF, the fracture must be located along the femoral diaphysis from just distal to the lesser trochanter to just proximal to the supracondylar flare. In addition at least four of five Major Features must be present. None of the Minor Features is required but have sometimes been associated with these features".
  - "Major features: The fracture is associated with minimal or no trauma, as in a fall from a standing height or less".
    - "The fracture line originates at the lateral cortex and is substantially transverse in its orientation, although it may become oblique as it progresses medially across the femur".
    - "Complete fractures extend through both cortices and may be associated with a medial spike, incomplete fractures involve only the lateral cortex".
    - "The fracture is non-comminuted or minimally comminuted".
    - "Localized periosteal or endosteal thickening of the lateral cortex is present at the fracture site (“beaking” or “flaring”)".
  - "Minor features: Generalized increase in cortical thickness of the femoral diaphysis".
    - "Unilateral or bilateral prodromal symptoms such as dull or aching pain in the groin or thigh".
    - "Bilateral incomplete or complete femoral diaphysis fractures".
    - "Delayed fracture healing".

Further associated findings are comorbid conditions (e.g. vitamin D deficiency, rheumatoid arthritis, hypophosphatasia) and use of drugs such as bisphosphonates, glucocorticoids and proton pump inhibitors [10].

Atypical stress fractures are not common. Only 7–10 % of femoral fractures are located below the lesser trochanter; of these 75 % are due to major trauma reducing the incidence to 2-3 %.

**Fig. 7:**
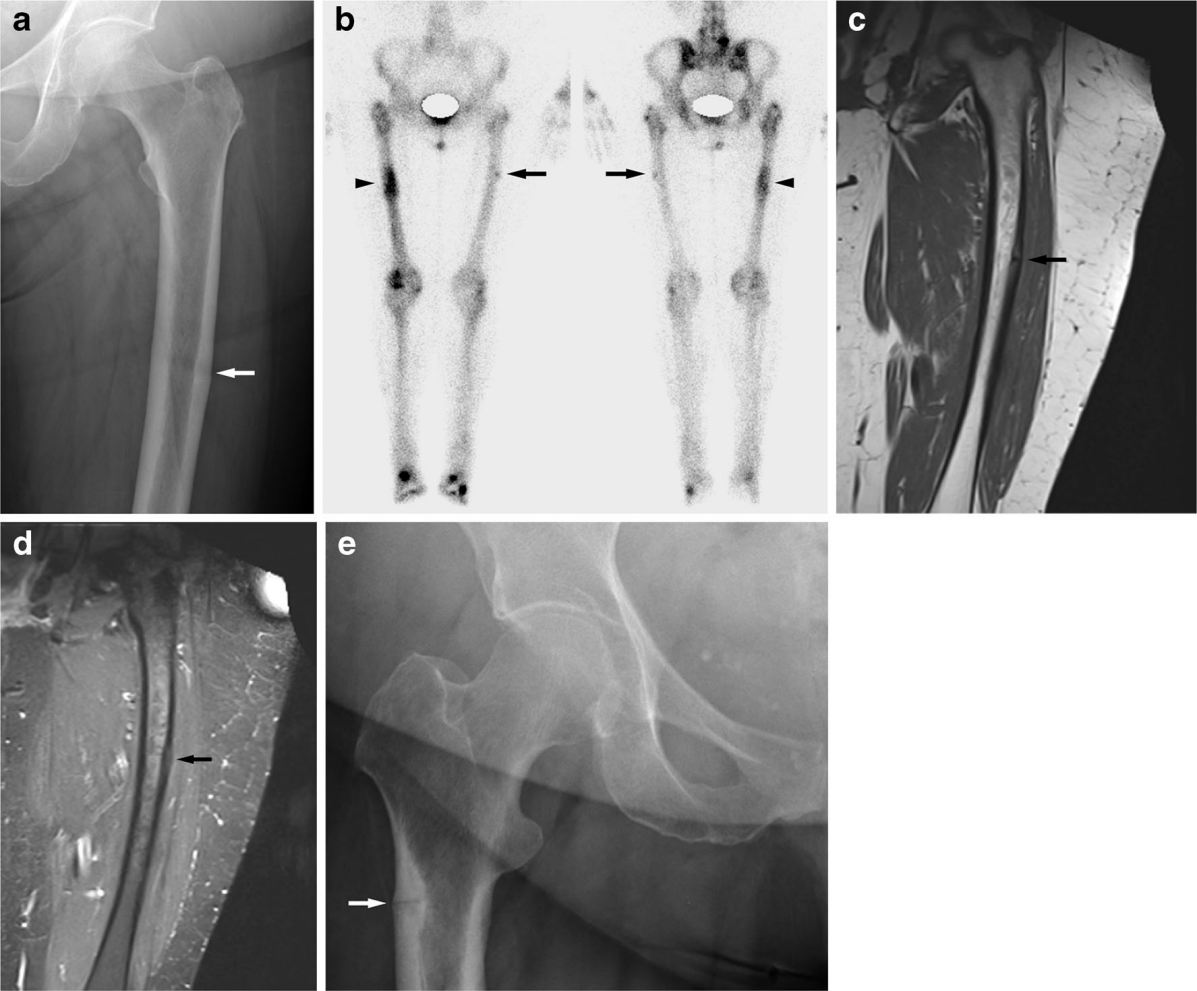
An elderly female on long-term bisphosphonates for osteoporosis had suffered a sudden fracture of the right femoral shaft after a period of niggling right thigh pain. This had been treated with an intramedullary nail. Then similar pain developed on the left side. A radiograph (**a**) shows subtle cortical thickening of the lateral aspect of the femoral shaft (see arrow). Bone scintigraphy (**b**) and MRI performed 1 month later (coronal T1w **c**, coronal STIR **d**) show mild uptake of the left femoral shaft and a minor bone reaction of the left lateral femoral shaft (see arrows). Marked uptake of the right femoral shaft on the bone scan is due to previous fracture; an intramedullary nail is in situ resulting in increased uptake in the proximal femur (see arrowhead). This is a so-called atypical femoral fracture. An AP radiograph of the hips performed for right hip/groin pain in another elderly female patient shows a more advanced case with a fracture through the lateral subtrochanteric femoral shaft cortex with a minor bone reaction; only a small bony spike is seen (**e**, arrow). Atypical femoral shaft fractures often affect elderly patients on long-term bisphosphonates. The lateral femoral cortex is affected first, bone reaction is minor, and the fracture lines are fairly horizontal

The differentiation of atypical femoral fractures from classic stress fractures is not always easy. “Normal” stress fractures would also produce fractures and a periosteal reaction. However stress fractures seen in athletes affect the medial cortex in the proximal third of the femoral shaft. The fracture line is more oblique than usually seen in atypical fractures. Atypical femoral shaft fractures primarily affect the lateral cortex and the atypical fractures are associated with more generalised cortical thickening rather than just focal change (Figs. 4 and 7).

The atypical fractures are thought to be due to abnormally brittle bone. Brittle bone is thought to be susceptible to fracture because of high tensile forces on the lateral femoral cortex affecting the lateral aspect of the femoral shaft. This theory is supported by biomechanical analysis and the distribution of the atypical fractures along the femoral shaft, which correlates with the angulation of the femur against the tibia; higher angulation results in a more caudal position of the fracture along the femoral shaft [10, 28]. The cortical medial bone spike that may be seen in atypical femoral fractures is a focal bone response to the developing fracture. It is very focal and dense; a periosteal reaction is not typically seen.

Subtrochanteric fractures have been reported as having a 14 % mortality at 12 months and 25 % mortality at 24 months. This makes it a clinically and prognostically relevant diagnosis [10]. The high mortality of subtrochanteric fractures is not unique to atypical femoral fractures. Extracapsular femoral neck fractures and femoral shaft fractures result in a higher mortality and morbidity than intracapsular femoral neck fractures [29, 30].

It has been suggested that some (and possibly most) of these fractures are due to bisphosphonate use. In 310 cases with the diagnosis of atypical femoral fracture, bisphosphonate use was found in 291, mostly for treatment of osteoporosis (286/310) and a few (5/310) for treatment of bone metastases. In 28 % of cases bilateral atypical femoral fracture or radiographic abnormalities were found. Atypical femoral fracture may also be seen in other conditions associated with abnormal bone structures such as hypophosphatasia, pycnodysostosis or osteopetrosis [28].

The exact aetiology of the particular condition is not clear. It is assumed that a decrease in bone remodelling as well as changes in collagen maturity and collagen crosslinking and possibly altered angiogenesis are responsible. Changes in the collagen make-up affect its mechanical strength and elasticity and also the strength and elasticity of other structures such as bone. Bisphosphonate use increases the risk of affecting the collagen make-up. Nevertheless it is clear that the benefit of bisphosphonate use (reduction in vertebral and femoral neck fractures) by far outweighs the risk of atypical femoral fractures [10, 28, 31].

For the radiologist it is important to be familiar with this pathology because this diagnosis should trigger specialist referral. A patient with this diagnosis should not be on bisphosphonates and femoral pinning might be required though the individual circumstances need to be carefully assessed. The authors are aware of cases where atypical femoral fractures were misdiagnosed as “normal” insufficiency fractures leading to continued or even newly instigated bisphosphonate treatment and eventually to overt fracture.

### Migrating bone marrow oedema syndrome, transient osteoporosis, transient bone marrow oedema syndrome, spontaneous osteonecrosis of the knee (SONK)

MR imaging of joints not rarely shows areas of increased fluid signal immediately adjacent to the joint or in the proximity. While some of these signal changes can be explained by degenerative changes, larger, ill-defined areas of oedema-like signal cannot. It was recognised that the radiographic diagnosis of transient osteoporosis has an MRI equivalent and indeed that areas of diffuse oedema-like signal can show different imaging and clinical outcomes with time.

Large ill-defined areas of bone marrow oedema-like change can spontaneously resolve without associated clinical or imaging findings. They can be associated with pain or radiographically visible transient osteoporosis before resolution occurs. The oedema-like signal changes might show small crescent-shaped areas of subchondral contour concavity and sclerosis and this is now thought to represent subchondral stress fractures. This is seen particularly on the convex joint surface of large joints, especially the femoral head and femoral condyles. The overlying cartilage is intact here (Fig. 6).

Areas of osteonecrosis might also develop on the background of bone marrow oedema-like change [32, 33].

This is distinct from the MRI findings seen in avascular necrosis (avn). In cases of avn disruption of the blood supply to the bone is the primary pathology. The bone marrow shows signal changes with typical serpiginous margins. With ischaemia bone necrosis occurs but in avn necrosis is due to primary disruption of the blood supply as opposed to a primary mechanical bone injury in stress fractures. Bone necrosis due to any cause can lead to bone collapse/fracture. The serpiginous morphology sets primary avn apart from other pathologies and can usually be identified. Often avn is due to a generalised metabolic problem and other sites in the body may show typical findings in cases where local collapse has occurred [34, 35].

It is important to realise that what appears like increased fluid signal on MRI and is therefore colloquially called “bone marrow oedema” is in fact granulation tissue with fibrovascular ingrowth [36]. Oedema-like bone marrow changes therefore represent a tissue reaction to a variety of stimuli; trabecular microfractures leading to bone repair is one of them.

Over the years a number of theories have been brought forward to explain transient or permanent bone marrow signal change around weight-bearing joints. Venous congestion, synovitis and atraumatic reflex sympathetic dystrophy have all been proposed and largely refuted. Yamamoto and Bullough have proposed stress (insufficiency) type fractures as the cause for these syndromes and this theory could explain the range of imaging findings that might or might not progress to more serious presentations including osteonecrosis or even rapid osteolysis of the femoral head [37–40].

**Fig. 8:**
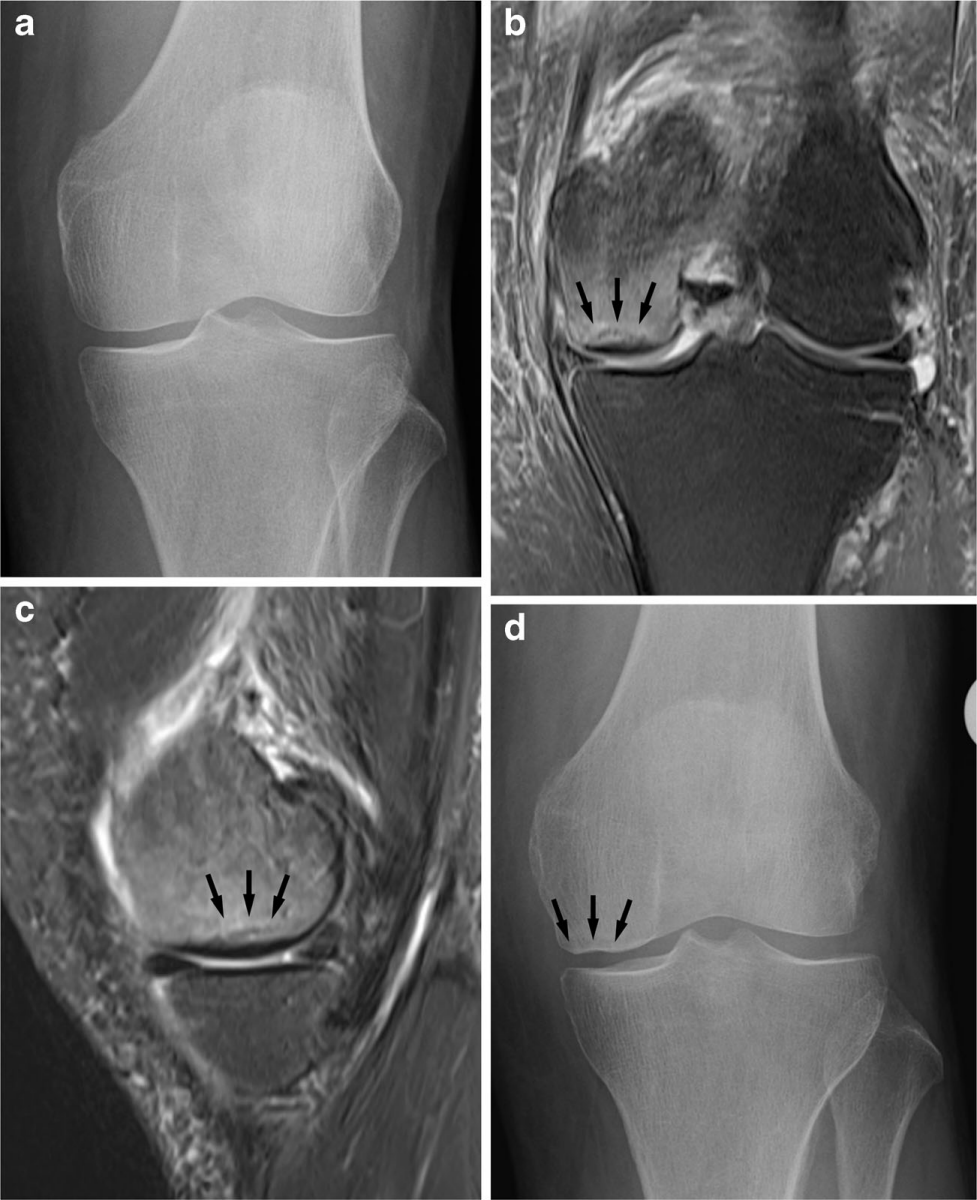
An elderly female with sudden spontaneous onset of pain. Initial radiographs (shown AP view, **a**) do not show a focal anomaly. MRI 1 month later performed for persistent pain shows extensive bone marrow oedema-like change in the medial femoral condyle with a subchondral sclerotic area consistent with an insufficiency fracture (**b**, **c**; PD-FS; see arrows). The features are those of spontaneous osteonecrosis of the knee, SONK. Note the degeneration/tear of the medial meniscus, a common association of SONK. Radiographic follow-up 1 month after the MRI shows partial cortical collapse of the medial femoral condyle (**d**, see arrows)

Applied to the knee spontaneous osteonecrosis of the knee (SONK) can be thought of as a stress fracture of subchondral bone. Any feature increasing the mechanical stress onto bone, such as meniscal degeneration, or weakening bone, such as osteopenia, should and do lead to an increased risk of SONK (Fig. 8). With any significant bone injury some bone tissue death, necrosis, will occur. In the case of SONK this is secondary to mechanical stress and thus “encouraged” by any condition weakening bone or increasing bone stress [32, 33, 37, 39, 41–43].

Some of the bone marrow changes identified on MRI are of course incidental findings and resolve spontaneously and never have associated clinical symptoms. This phenomenon is already known from bone scintigraphy studies of athletes [7, 20, 32].

### Stress fractures after radiotherapy

Stress fractures after radiotherapy are not rare and often pose a diagnostic challenge to differentiate stress fractures from metastatic disease.

Pelvic stress fractures after radiotherapy are well recognised. Common primary pathologies are cervical cancer and rectal cancer. The fracture risk is significantly higher for females than for males and generally is higher for higher radiation doses. At dose levels of less than 40 Gy to the pelvis radiation-induced stress fractures are uncommon. Above 40 Gy fractures occur in increasing frequency with increasing dose. A recent review quoted a cumulative incidence of pelvic insufficiency fracture after radiotherapy in females as 13 % over 5 years with about ¾ of these occurring in the first year and most within the first 2 years [44]. Incidences in individual studies varied widely with one study quoting a fracture incidence of up to 89 % [44–46]!

The lower limbs are less well known sites for post-radiotherapy stress fractures. Radiotherapy here was usually administered for the treatment of soft tissue sarcomas. Similar to the findings in the pelvis the risk for post-radiotherapy fracture is higher for higher dose treatment regimes and in females, particularly those over the age of 55 years. Preoperative radiation treatment resulted in a significantly lower incidence of stress fractures when compared with postoperative radiotherapy (roughly lower by a factor 10) but leads to more wound complications after surgery [46].

Radiotherapy leads to direct cell death and also damages microvascular structures leading to depletion of red marrow and proliferation of fatty marrow. After an initial phase of bone marrow oedema lasting just a few weeks, the fatty marrow conversion takes place. Soft tissue oedema after radiotherapy is often longer lasting. Radiotherapy can lead to direct bone necrosis; usually this is incomplete and a reactive inflammatory reaction ensues. This frequently leads to sclerotic bone change of the trabeculae and cortex with reduced mechanical strength and repair capacity. This in turn makes the bone vulnerable to stress fractures [44, 45, 47].

**Fig. 9:**
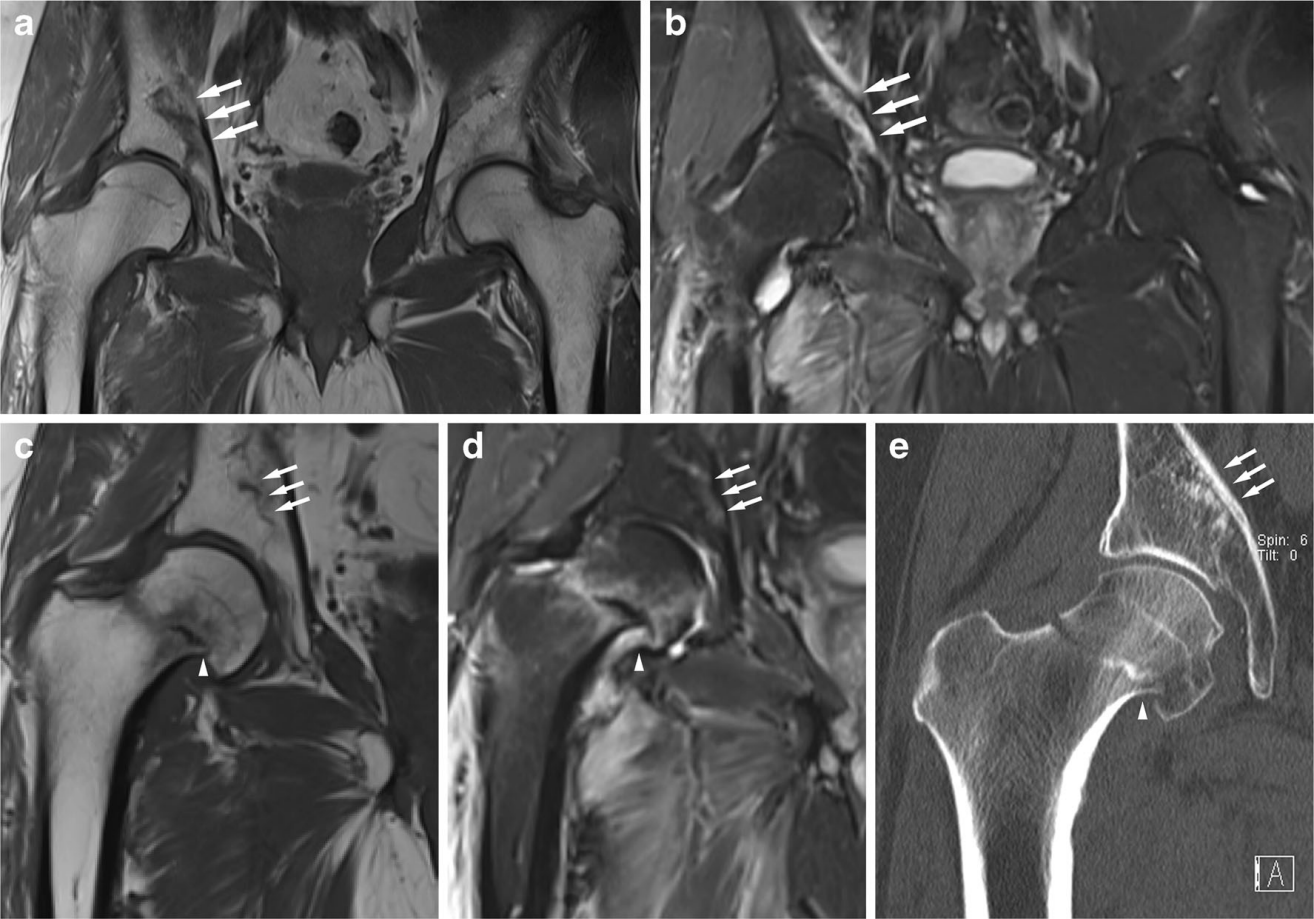
A male patient in his early 60s underwent surgical resection and postsurgical radiotherapy of the right groin and proximal femur for a soft tissue sarcoma. Five months after the radiotherapy the patient experienced right groin pain, and local recurrence was suspected. MRI showed a severe stress fracture of the right acetabulum [coronal T1w (**a**) and coronal STIR (**b**) images; see arrows]. Eleven months after the radiotherapy the patient developed a spontaneous right femoral neck fracture after prodromal pain. Coronal MRI (**c** T1w, **d** STIR) shows the absence of an underlying malignancy, an impacted femoral neck fracture (arrowhead) and bone marrow with high fat content. The stress fracture of the acetabular roof has largely healed (arrows). There is persistence of marked soft tissue oedema after radiotherapy. CT imaging (**e**, coronal reformat) also demonstrates the femoral neck fracture (arrowhead) and shows mild sclerosis of the bone marrow in the femoral head and neck, and no obvious osteopenia. The acetabular stress fracture can be appreciated as an area of sclerosis (arrows). Radiotherapy impairs the bone repair capability and predisposes the patient to stress fractures, often in the first year after radiotherapy

The best single imaging modality to assess focal bone abnormalities after radiotherapy is MRI as it combines good sensitivity and specificity. Diffusion-weighted imaging might help to differentiate a stress fracture from bone metastases and pathological fracture. MRI typically demonstrates oedema-like changes typical of insufficiency fractures. CT is also often useful and able to demonstrate abnormal bone texture and fractures. Bone marrow change is of course difficult to assess with CT. Radiographs often are of limited use and bone scintigraphy has good sensitivity but limited specificity (Fig. 9) [44].

The biggest challenge in case of pelvic fractures after radiotherapy is usually the differentiation between a benign stress fracture and metastasis. In cases of stress fractures in the pelvis the sacrum is usually involved first. Stress fractures are often bilateral and also symmetrical and they are confined to the field of irradiation. The bone marrow oedema-like change is usually extensive and surrounding soft tissue masses or oedema limited. Actual fracture lines are seen and diffusion-weighted MRI should show low signal because of unrestricted diffusion.

In contrast metastatic disease with pathological fractures can occur anywhere in the pelvis and there is no symmetry and no confinement to the field of irradiation. Bone marrow signal change is limited and soft tissue involvement is more common. Diffusion-weighted MRI often shows high signal due to restricted diffusion [44, 45].

### Stress fractures by area

#### Pelvis

The bony pelvis has several well-known sites of predilection for stress fractures. Most doctors will be familiar with sacral and pubic fractures. Sacral stress fractures can be difficult to identify on radiographs but are usually straightforward on MRI, CT and bone scintigraphy. A typical H-shape with vertical fractures through the sacral ala and a horizontal fracture through the body of the sacrum is often (but not always) seen.

**Fig. 10:**
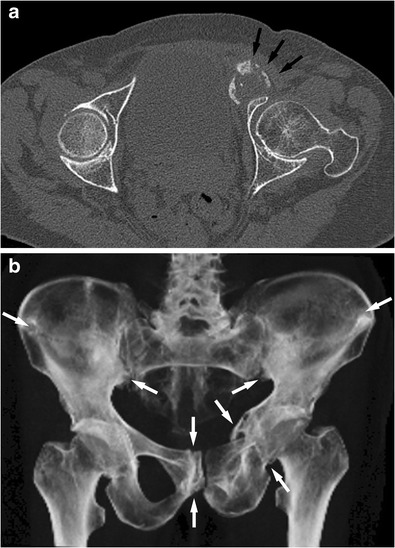
CT of the pelvis of an elderly female with multiple stress fractures due to osteoporosis. The axial images of the fractures could be mistaken for an aggressive destructive process (**a**, arrows) showing an apparent expansile bone lesion anterior to the acetabulum. However a volume-rendered image (**b**) of the pelvis shows multiple and partly symmetrical fractures typical for severe insufficiency fractures. Fractures of the iliac blades, the right os pubis and left pubic rami can be seen (see arrows). The axial images in isolation may be difficult to interpret; 3D reformats and volume rendering help in image interpretation

Further common sites for stress fractures in the pelvis are the pubic rami, often identifiable radiographically as healed fractures, and the symphysis pubis, here leading to irregular sclerosis. Fracture of the pubic rami can lead to quite marked focal bone lysis, which can be difficult to differentiate from a destructive malignancy. The identification of either further stress fractures or stress-related bone lesions or further lytic destructive lesions usually helps to make the correct diagnosis (Fig. 10).

Fractures of the iliac wings usually occur in more severe cases of insufficiency type fractures.

Fractures of the acetabular roof are also usually true insufficiency fractures rather than fatigue type fractures (Fig. 9).

Generally pelvic stress fractures are mostly due to insufficiency fractures and assessment of bone metabolism is usually indicated.

The pelvis is also a common site for stress fractures after radiotherapy. The commonly seen background change of bone sclerosis can cause concern regarding an underlying bone-forming malignancy. A relevant clinical history or identification of a radiation portal with relatively sharp outlines can help to make the correct diagnosis [48].

On the whole pelvic stress fractures have a low risk of non-union [4].

#### Femur

The proximal femur can suffer stress fractures in a number of sites. Femoral neck fractures are perhaps best known but they can be radiographically subtle in the early stages, and in activity-related pain with negative radiographs further imaging workup with MRI is indicated. The typical femoral neck stress fracture is located on the medial aspect and perpendicular to the stress lines and the femoral neck (Fig. 3). They are due to compressive forces and seen more in younger, more athletic patients. Femoral neck fractures on the craniolateral aspect of the femoral neck are more common in older patients. These fractures are due to distraction; the risk of non-union is higher than for the medial compression fractures although for both types the risk of non-union is relatively high compared with other sites [13, 14, 16, 19].

Subchondral stress fractures of the femoral head are harder to detect. Radiographically there might be subtle contour concavities of the weight-bearing femoral head and subcortical zones of sclerosis and lucencies (Fig. 6). These findings are often due to insufficiency fractures and the femoral head might be at risk of sudden collapse [49, 50].

It has been suggested that transient osteoporosis of the hip might be due to stress fractures.

Stress fractures may further occur at the greater and the lesser trochanter.

The subtrochanteric femoral shaft can be affected by typical and atypical stress fractures. As discussed in a paragraph above, atypical femoral shaft fractures typically affect the lateral femoral cortex with very focal changes while typical femoral shaft stress fractures in athletes usually affect the medial cortex with a more broad-based periosteal reaction (Figs. 4 and 7) [51].

#### Knee

Around the knee stress fractures may affect the femoral condyles particularly subchondrally. The pathology of spontaneous osteonecrosis of the knee, SONK, has also been suggested to be due to stress fractures (Fig. 8) [33, 41–43].

Patella and tibia plateau stress fractures are more commonly due to insufficiency fractures. In particular transverse patella fractures have a high risk of becoming complete fractures. Transverse stress fractures are due to traction stress; longitudinal stress fractures of the patella are less common and are due to compression against the femoral condyles [13, 16].

#### Tibia and fibula

**Fig. 11:**
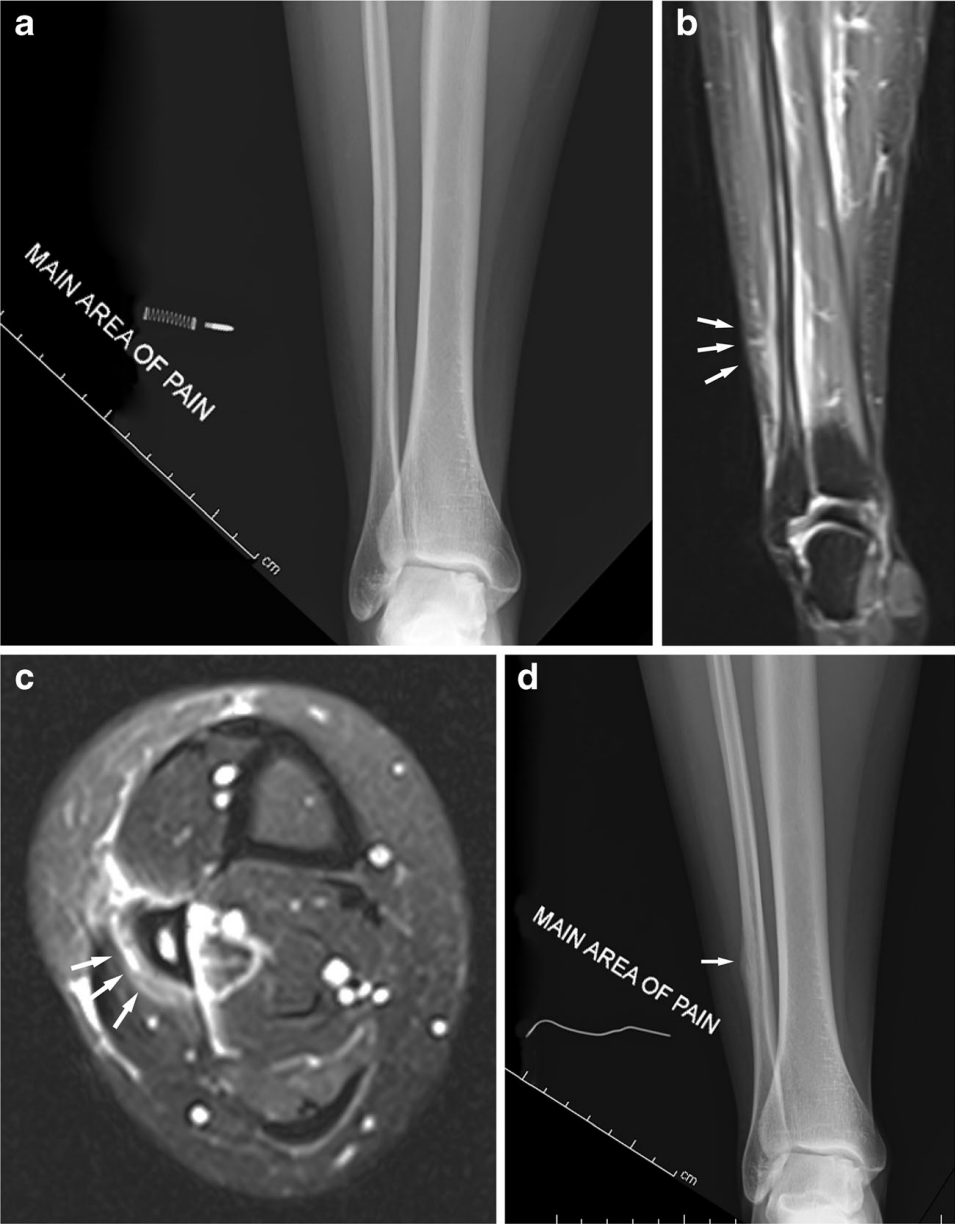
Middle-aged female runner presenting with focal calf pain. Initial radiographs (**a**, AP view shown) were normal. MRI 2 weeks after the initial radiographs (**b**, **c**, coronal and axial PD-FS images) shows a periosteal reaction and widespread soft tissue oedema around the fibula shaft typical for a stress fracture (see arrows). Radiographic follow-up 3 weeks after this (**d**) shows a periosteal reaction of the fibula where pain was initially indicated (arrow). Radiographs are insensitive for stress fractures early on; MRI shows soft tissue oedema and a periosteal reaction and is the investigation of choice if radiographs are negative

The tibia is a common site for stress fractures and some authors suggest it is the most common site overall [4]. Compression type stress fractures typically affect the posteromedial cortex and usually heal with rest. Longitudinal stress fractures are less common but still heal well with rest. Tension stress fractures typically affect the anterior, convex cortex, and this injury can be slow to heal and more commonly go on to become complete fractures. CT can be very useful for imaging of tibial stress fractures. Fibular stress fractures present in a similar fashion. They are not rare and may affect the fibular head, shaft or lateral malleolus (Figs. 5 and 11) [4, 13].

#### Ankle/hindfoot/midfoot

In the ankle and hindfoot area, stress fractures can affect the calcaneus, distal fibula, talar trochlea, calcaneus and navicular. All these sites are commonly affected. Calcaneal fractures are often seen in osteopenic patients. The diagnosis can be difficult radiographically. Sclerotic lines may be seen in the dorsal aspect of the calcaneus. They are often parallel to the cortex and here perpendicular to the main orientation of the trabecular bone; thus they can be differentiated from normal trabeculae. If in doubt MRI is diagnostic.

Calcaneal and distal fibular fractures usually heal well. Talar and navicular fractures are at high risk for delayed healing and even progression to avascular necrosis and collapse due to the pattern of vascular supply, which can be disrupted with typical proximal stress fractures.

Medial malleolar fractures are not common but if they occur have a high risk of non-union (Fig. 5) [7, 13, 16].

#### Forefoot

In the appendicular skeleton and pelvis the forefoot is the area most commonly affected by stress fractures, particularly metatarsal stress fractures. The shafts of the second and thirrd metatarsal are most commonly affected. These usually heal well with appropriate rest (Figs. 1 and 2). If the base of the fifth metatarsal is affected there is a significant risk of non-union and surgical treatment may be indicated. Stress fractures of the sesamoids of the big toe are also associated with a high risk for non-union [3, 6, 7, 13, 16, 52].

## Conclusion

Stress fractures of the pelvis and lower limbs can be insidious in onset and hard to diagnose if there is overreliance on radiographic abnormalities. If there is sufficient clinical suspicion MRI or bone scintigraphy is the imaging modality of choice. Fatigue and insufficiency mechanisms can be interrelated and their differentiation is not always easy or possible. Recognition of atypical femoral shaft fractures is important and review by a metabolic physician is indicated to optimise patient treatment and outcome.

## References

[1] Breithaupt MB (1855). Zur Pathologie des Menschlichen Fusses. Med Z.

[2] Stechow AW (1897). Fussoedem und Roentgenstrahlen. Dtsch Mil Aerztl Z.

[3] Nampei A, Hashimoto J, Koyanagi J (2008). Characteristics of fracture and related factors in patients with rheumatoid arthritis. Mod Rheumatol.

[4] Ahn JM, El-Khoury GY, Pope B, Morrison B, Wilson (2008). Stress Injury, in Imaging of the Musculoskeletal System.

[5] Patel DS, Roth M, Kapil N (2011). Stress fractures: diagnosis, treatment, and prevention. Am Fam Physician.

[6] Breer S, Krause M, Marshall RP (2012). Stress fractures in elderly patients. Int Orthop.

[7] Fredericson M, Wun C (2003). Differential diagnosis of leg pain in the athlete. J Am Podiatr Med Assoc.

[8] Edwards MR, Jack C, Jones GG (2010). Post-operative stress fractures complicating surgery for painful forefoot conditions. Foot (Edinb).

[9] Ito T, Jensen RT (2010). Association of long-term proton pump inhibitor therapy with bone fractures and effects on absorption of calcium, vitamin B12, iron, and magnesium. Curr Gastroenterol Rep.

[10] Shane E, Burr D, Ebeling PR (2010). Atypical subtrochanteric and diaphyseal femoral fractures: report of a task force of the American Society for Bone and Mineral Research. J Bone Miner Res.

[11] Chouhan V, Agrawal K, Vinothkumar TK (2010). Bilateral insufficiency fracture of the femoral head and neck in a case of oncogenic osteomalacia. J Bone Joint Surg Br.

[12] Kanis JA, McCloskey EV, Johansson H (2010). Development and use of FRAX in osteoporosis. Osteoporos Int.

[13] Berger FH, de Jonge MC, Maas M (2007). Stress fractures in the lower extremity. The importance of increasing awareness amongst radiologists. Eur J Radiol.

[14] Bousson V, Wybier M, Petrover D (2011). Les fracture de contrainte [Stress fractures]. J Radiol.

[15] Krestan C, Hojreh A (2009). Imaging of insufficiency fractures. Eur J Radiol.

[16] Fredericson M, Jennings F, Beaulieu C (2006). Stress fractures in athletes. Top Magn Reson Imaging.

[17] Matheson GO, Clement DB, McKenzie DC (1987). Stress fractures in athletes. A study of 320 cases. Am J Sports Med.

[18] Groves AM, Cheow HK, Balan KK (2005). 16-Detector multislice CT in the detection of stress fractures: a comparison with skeletal scintigraphy. Clin Radiol.

[19] Drubach LA, Connolly LP, D’Hemecourt PA (2001). Assessment of the clinical significance of asymptomatic lower extremity uptake abnormality in young athletes. J Nucl Med.

[20] Matheson GO, Clement DB, McKenzie DC (1987). Scintigraphic uptake of 99mTc at non-painful sites in athletes with stress fractures. The concept of bone strain. Sports Med.

[21] Bergman AG, Fredericson M, Ho C (2004). Asymptomatic tibial stress reactions: MRI detection and clinical follow-up in distance runners. AJR Am J Roentgenol.

[22] Tins B, Cassar-Pullicino V (2006). Marrow changes in anorexia nervosa masking the presence of stress fractures on MR imaging. Skeletal Radiol.

[23] Banal F, Gandjbakhch F, Foltz V (2009). Sensitivity and specificity of ultrasonography in early diagnosis of metatarsal bone stress fractures: a pilot study of 37 patients. J Rheumatol.

[24] Howard CB, Lieberman N, Mozes G (1992). Stress fracture detected sonographically. AJR Am J Roentgenol.

[25] Arni D, Lambert V, Delmi M (2009). Insufficiency fracture of the calcaneum: Sonographic findings. J Clin Ultrasound.

[26] Bodner G, Stockl B, Fierlinger A (2005). Sonographic findings in stress fractures of the lower limb: preliminary findings. Eur Radiol.

[27] Drakonaki EE, Garbi A (2010). Metatarsal stress fracture diagnosed with high-resolution sonography. J Ultrasound Med.

[28] Shane E, Burr D, Abrahamsen B et al (2013) Atypical subtrochanteric and diaphyseal femoral fractures: second report of a task force of the American Society for Bone and Mineral Research. J Bone Miner Res 10.1002/jbmr.1998 23712442

[29] Abrahamsen B, van Staa T, Ariely R (2009). Excess mortality following hip fracture: a systematic epidemiological review. Osteoporos Int.

[30] Keene GS, Parker MJ, Pryor GA (1993). Mortality and morbidity after hip fractures. BMJ.

[31] Edwards BJ, Bunta AD, Lane J (2013). Bisphosphonates and nonhealing femoral fractures: analysis of the FDA Adverse Event Reporting System (FAERS) and international safety efforts: a systematic review from the Research on Adverse Drug Events And Reports (RADAR) project. J Bone Joint Surg Am.

[32] Gil HC, Levine SM, Zoga AC (2006). MRI findings in the subchondral bone marrow: a discussion of conditions including transient osteoporosis, transient bone marrow edema syndrome, SONK, and shifting bone marrow edema of the knee. Semin Musculoskelet Radiol.

[33] Korompilias AV, Karantanas AH, Lykissas MG (2009). Bone marrow edema syndrome. Skeletal Radiol.

[34] Lafforgue P (2006). Pathophysiology and natural history of avascular necrosis of bone. Joint Bone Spine.

[35] Schmidt GP, Reiser MF, Baur-Melnyk A (2009). Whole-body imaging of bone marrow. Semin Musculoskelet Radiol.

[36] Zanetti M, Bruder E, Romero J (2000). Bone marrow edema pattern in osteoarthritic knees: correlation between MR imaging and histologic findings. Radiology.

[37] Yamamoto T, Bullough PG (2000). Spontaneous osteonecrosis of the knee: the result of subchondral insufficiency fracture. J Bone Joint Surg Am.

[38] Yamamoto T, Schneider R, Bullough PG (2000). Insufficiency subchondral fracture of the femoral head. Am J Surg Pathol.

[39] Yamamoto T, Bullough PG (2000). Subchondral insufficiency fracture of the femoral head and medial femoral condyle. Skeletal Radiol.

[40] Yamamoto T, Bullough PG (2000). The role of subchondral insufficiency fracture in rapid destruction of the hip joint: a preliminary report. Arthritis Rheum.

[41] Yao L, Stanczak J, Boutin RD (2004). Presumptive subarticular stress reactions of the knee: MRI detection and association with meniscal tear patterns. Skeletal Radiol.

[42] Ramnath RR, Kattapuram SV (2004). MR appearance of SONK-like subchondral abnormalities in the adult knee: SONK redefined. Skeletal Radiol.

[43] Akamatsu Y, Mitsugi N, Hayashi T (2012). Low bone mineral density is associated with the onset of spontaneous osteonecrosis of the knee. Acta Orthop.

[44] Meerveld-Eggink A, Bollen TL, Wijrdeman HK (2013). Bone metastases or an insufficiency fracture? Oncology patients reporting pain or showing bone abnormalities on a scan. Ned Tijdschr Geneeskd.

[45] Moreno A, Clemente J, Crespo C (1999). Pelvic insufficiency fractures in patients with pelvic irradiation. Int J Radiat Oncol Biol Phys.

[46] Holt GE, Griffin AM, Pintilie M (2005). Fractures following radiotherapy and limb-salvage surgery for lower extremity soft-tissue sarcomas. A comparison of high-dose and low-dose radiotherapy. J Bone Joint Surg Am.

[47] Georgiou KR, Hui SK, Xian CJ (2012). Regulatory pathways associated with bone loss and bone marrow adiposity caused by aging, chemotherapy, glucocorticoid therapy and radiotherapy. Am J Stem Cells.

[48] Hosono M, Kobayashi H, Fujimoto R (1997). MR appearance of parasymphseal insufficiency fractures of the os pubis. Skeletal Radiol.

[49] Boutry N, Paul C, Leroy X (2002). Rapidly destructive osteoarthritis of the hip: MR imaging findings. AJR Am J Roentgenol.

[50] Davies M, Cassar-Pullicino VN, Darby AJ (2004). Subchondral insufficiency fractures of the femoral head. Eur Radiol.

[51] Nachtrab O, Cassar-Pullicino VN, Lalam R (2012). Role of MRI in hip fractures, including stress fractures, occult fractures, avulsion fractures. Eur J Radiol.

[52] Nwawka OK, Hayashi D, Diaz LE et al Sesamoids and accessory ossicles of the foot: anatomical variability and related pathology. Insights Imaging 4(5):581–593 10.1007/s13244-013-0277-1 PMC3781258 24006205

